# Identification of the Risk Factors for the Failure of Ureteral Access Sheath Placement

**DOI:** 10.1155/2022/7518971

**Published:** 2022-09-05

**Authors:** Jieping Hu, Yue Yu, Wei Liu, Jialei Zhong, Xiaochen Zhou, Haibo Xi

**Affiliations:** Department of Urology, The First Affiliated Hospital of Nanchang University, Nanchang, China

## Abstract

**Purpose:**

Insertion of a ureteral access sheath (UAS) may fail in some patients in retrograde intrarenal surgery (RIRS), and this study aimed to seek preoperative risk factors for the failure of 12/14F UAS placement.

**Methods:**

We retrospectively analyzed 260 consecutive patients who underwent RIRS between May 2020 and March 2022 at our institution. Data on patient and stone characteristics and several computed tomography (CT)-based measurements were collected and compared between the success and failure UAS placement groups.

**Results:**

Twenty-nine (11.2%) patients failed to insert the UAS. Age, gender, height, weight, stone side, stone location, length of history, and computed tomography (CT)-based parameters were not significant differences between the two groups. Univariate logistic regression analyses showed sex (female/male) (odds ratio: 0.287 and 95% CI [0.107, 0.722], *p*=0.013), length of history 15–31 days (odds ratio: 0.315 and 95% CI [0.102, 0.974], *p*=0.045), length of history >31 days (odds ratio: 0.202 and 95% CI [0.051, 0.805], *p*=0.023), and diameter of the ipsilateral common iliac artery (odds ratio: 1.285 and 95% CI [1.018, 1.623], *p*=0.035) were associated with UAS placement.

**Conclusion:**

Our study indicated that males, the short length of history, and the short diameter of the ipsilateral common iliac artery were the risk factors for the failure of UAS placement.

## 1. Introduction

Retrograde intracranial surgery (RIRS) is a treatment option for renal or proximal ureteral stones [1]. Ureteral access sheaths (UAS) increase visibility, reduce operating time, and allow multiple reentries to the ureter and are widely used in RIRS [2]. However, failure rates of ureteroscopy due to a difficult impassable ureter range from 8% to 10%, and approximately 22% of patients fail to insert a standard UAS [3]. Stent placement before RIRS can theoretically expand the ureter to improve access, and data showed prestenting resulted in higher success for UAS placement and also minimized intraoperative ureteric injury [1, 4]. In vitro experiments showed that one week of ureteral stenting resulted in nearly a 4Fr increase in the luminal circumference of porcine ureters [5]. Nevertheless, European Association of Urology (EAU) guidelines did not suggest the routine placement of ureteral stents before RIRS [4, 6]; it is of significant importance to identify risk factors for the failure of ureteral access sheath placement.

The size of the UAS has impacted the success of UAS insertion, smaller diameter UASs (<12/14 Fr) decrease the risk of ureteral wall injury, and larger diameter UASs (>12/14 Fr) are observed to improve surgical efficiency at the cost of greater risks of placement failures [7]. The 12/14 access sheath was found to be the device that accepts all available flexible ureteroscopes [8], and the ureter diameter on the asymptomatic side was 3 mm or smaller when evaluating patients with acute flank pain and suspected ureterolithiasis by computer tomography; the mean size of ureters on the obstructed side was 7 mm with an SD of 3.2 mm [9], and these lead to that 12/14 UAS which was mostly used in practice. The previous study indicated that age, previous same-side procedures, and preoperative stent were indicated to be independent predictors for an effective 12/14F UAS insertion [3], and others raised that patients with normal body mass index (BMI) and a tent-shaped ureteral orifice over the guide wires were found to be more likely to insert UAS [10], while male gender and ipsilateral hydronephrosis may be associated with UAS placement failure [11]. Here, we aimed to seek preoperative risk factors for the failure of 12/14F UAS placement to identify the patients who were recommended to receive a preoperative ureteral stent.

## 2. Patients and Methods

### 2.1. Study Design and Participants

This retrospective study was approved by the institutional review board of the First Affiliated Hospital of Nanchang University, and it was exempted from obtaining informed consent. We conducted the trial under the principles of the ethical principles of the Declaration of Helsinki. We included adults aged ≥18 years old with stones confirmed by noncontrast computed tomography (CT) with a slice thickness of 1 mm. We retrospectively analyzed the medical records and CT of 260 consecutive patients who underwent RIRS between May 2020 and March 2022 at our institution. Patients were screened by the following inclusion and exclusion criteria. The inclusion criteria were as follows: 1. upper ureteral or renal calculus confirmed by CT; 2. the patient agreed to receive RIRS; 3. 12/14-Fr ureteral access sheath (UAS) (cat no: 90111240 for females, 90111246 for males, Well Lead Medical Co., Ltd) was used; 4. age ≥18 years. The exclusion criteria were as follows: 1. preoperative ureteral stenting; 2. abnormal urinary tract anatomy (such as horseshoe kidney or ileal conduit); 3. patients received RIRS under local anesthesia; 4.11/13-Fr, 14/16-Fr, or another size of UAS was used; 5. the patient had previously undergone ureterolithotomy; 6. balloon catheter dilation was performed; 7. failure to receive RIRS due to pyonephrosis; 8. patients received RIRS because of renal stones with ipsilateral middle or lower ureteral stones.

### 2.2. Surgical Technique

The surgery was performed in a lithotomy position under general anaesthesia by two expert surgeons. A hydrophilic 0.035-inch guidewire was initially introduced into the renal pelvis. A semirigid ureteroscope (8/9.8-Fr) was initially inserted into the ureter along with a guidewire to inspect the whole length of the ureter. For impacted stone, holmium laser lithotripsy was used to dredge the stone into the pelvis. If an initial passage of the ureteroscope was not achieved, fascial dilators were used to dilate the ureter, and surgery would be terminated with an indwelling double J stent or change to percutaneous nephrolithotomy after evaluating the ureter by using the ureteroscope (these patients would be marked as UAS placement failure). Procedures were performed with caution not to cause trauma to the ureter. The following operation was conducted according to our institutional protocols. Patients were stratified according to the results into two groups: the effective passage of 12/14-Fr UAS with or without a need for fascial dilator dilation (not including balloon dilation) and failure to pass the 12/14-Fr UAS (including failure to pass the ureteroscope).

### 2.3. Statistical Analyses

**Figure 1:**
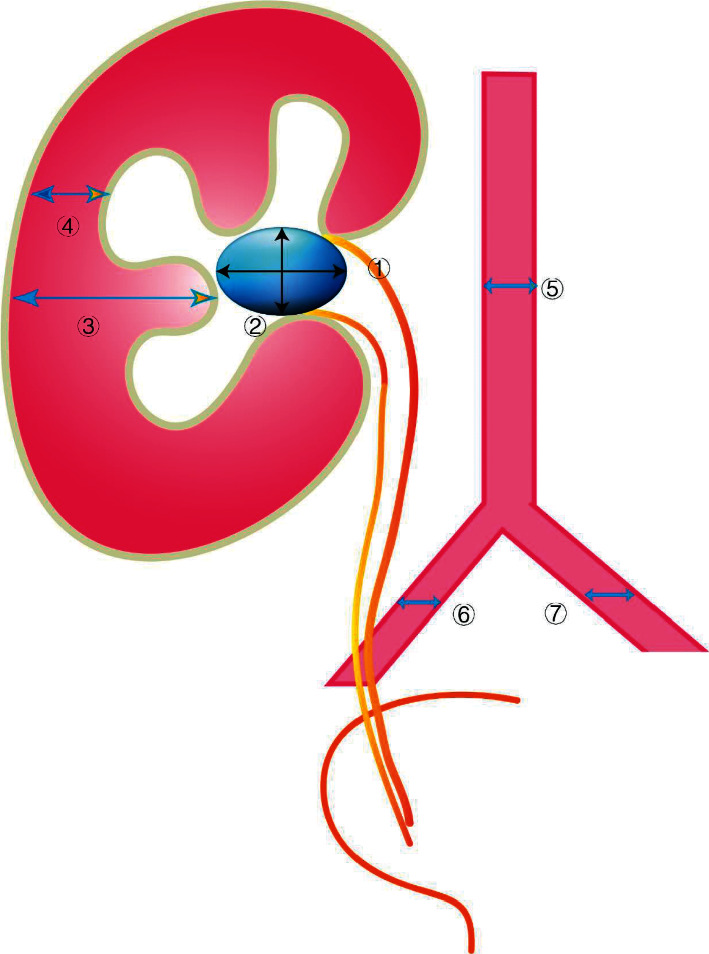
The computer tomography (CT)-based measurements contained seven parameters: ① long diameter of calculi; ② short diameter of calculi; ③ diameter of the widest part of the kidney parenchyma; ④ diameter of the narrowest part of the renal parenchyma; ⑤ diameter of the abdominal aorta; ⑥ diameter of the ipsilateral common iliac artery; ⑦ diameter of the contralateral common iliac artery.

Patient and stone characteristics, including age, gender, height, weight, length of history, stone size, side of stone, hydronephrosis, and several potential related CT-based measurements were collected. The length of history was defined as the time from onset to hospitalization. The length of history was divided into three categories: 0–14 days, 14–31 days, and more than 31 days. The CT-based measurements contained seven parameters: ① long diameter of calculi; ② short diameter of calculi; ③ diameter of the widest part of the kidney parenchyma; ④ diameter of the narrowest part of the renal parenchyma; ⑤ diameter of the abdominal aorta; ⑥ diameter of the ipsilateral common iliac artery; ⑦ diameter of the contralateral common iliac artery (Figure 1). The vascular diameter was assessed to identify whether it was related to the success of the ureteral sheath.

Continuous variables are expressed as mean values and standard deviations. Categorical variables are expressed as the frequencies of events (%). Pearson's chi-squared test and *T*-test were used for comparing the two groups. Factors affecting the success of UAS placement were analyzed using multivariate logistic regression. All statistical tests were performed using IBM SPSS Statistics, version 22.0 (IBM, Armonk, NY, USA), and a *p* value <0.05 indicated statistical significance.

## 3. Results

A total number of 260 patients were identified for analysis. Patient data are presented in Table 1. Twenty-nine (11.2%) patients failed to insert the ureteral access sheath (UAS) (group F), and two hundred and thirty-one patients were successful in inserting the UAS and performing RIRS (group S). Age, gender, height, weight, stone side, stone location, length of history, and computed tomography (CT)-based parameters were compared between the two groups, and there was no significant difference between the two groups.

**Figure 2:**
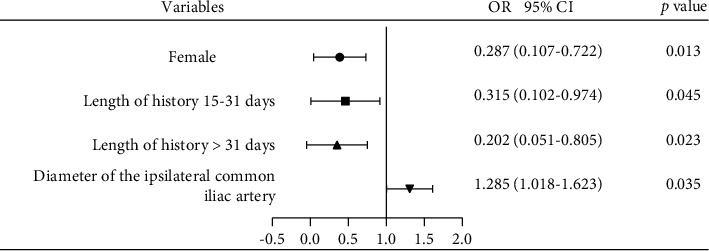
Risk factors for the failure of 14F ureteral access sheath (UAS) insertion.

Univariate logistic regression analysis was performed to identify the risk factors for the failure of ureteral access sheath placement, and sex (female/male) (odds ratio, OR: 0.287 and 95% CI [0.107, 0.722], *p*=0.013), length of history 15–31 days (OR: 0.315 and 95% CI [0.102, 0.974], *p*=0.045), length of history >31 days (OR: 0.202 and 95% CI [0.051, 0.805], *p*=0.023), and diameter of the ipsilateral common iliac artery (OR: 1.285 and 95% CI [1.018, 1.623], *p*=0.035) were found to be associated with UAS placement (Figure 2). Age, height, weight, stone side, stone location, and other parameters did not predict the failure of UAS insertion.

A subgroup analysis regarding upper ureteral or renal calculus, respectively, was conducted. Seventy-six patients had renal stones, six patients failed to insert UAS, and the short diameter of calculi was found to be associated with the success of UAS insertion (OR: 0.730 and 95% CI [0.556, 0.957], *p*=0.023). Of 184 patients who suffered from ureter stones, 23 patients failed to insert UAS, and none of the indexes were found to be associated with the failure of UAS insertion.

## 4. Discussion

The ureteral access sheath (UAS) is an important tool in the armamentarium of the endourologist and facilitates multiple and rapid passages of the ureteroscope during ureteroscopy. Our data indicated that males, the short length of history, and the short diameter of the ipsilateral common iliac artery were the risk factors for the failure of UAS placement. Larger diameter UASs (>12/14 Fr) allow for greater surgical efficacy and intrarenal pressure reduction to safe physiological levels at the cost of increased insertion forces, greater risk of ureteral wall injury, and lower insertion success rates. The use of a 12F/14F UAS in patients who are not previously stented increases the risk of high-grade ureteral injuries; however, despite this increase, there is no difference in ureteral stricture formation [12]. To our best knowledge, these factors were first reported to be associated with the insertion of a 12/14-Fr UAS. There are only a few pieces in the literature focused on the success of UAS placement [3, 10, 13]. Mogilevkin and others indicated that an indwelling double-J stent, a history of previous ureteroscopy or double-J stent, and older age were significant predictors for an effective 14F UAS insertion [3]. In our study, we found sex but not age was a possible predictor for UAS placement, which was similar to a previous study, and anatomical features of the urethra and ureter may be associated with this outcome [11]. Many studies have suggested that indwelling double-J stents result in higher success rates for UAS placement as ureteral stenting increases the luminal circumference of ureters [1, 4, 5, 14]. Patients who received preoperative ureteral stenting were not included in this study because EAU guidelines did not suggest routine placement of ureteral stents before RIRS [4, 6]. Alkhamees and others performed a study to identify the failure rate of insertion of a 10/12-Fr UAS, the outcomes were similar to our study, and in that, no statistically significant difference was found in age, BMI, and stone burden between the success and failure groups [13]. The ureter has 3 physiological strictures (pelvis renal, common iliac vessel crossing, and an intramural portion of the ureter). The intramural portion of the ureter could be one of the important factors for placement problems. Azhar and others prompted a new parament which was the configuration of the ureteral orifice (UO) over introductory guidewire insertion; they thought a tent-shaped UO was more likely to achieve 11/13-Fr UAS insertion compared with a round-shaped UO [10], and it was a good predictor, but the predictive value was limited as the shape could not be identified before surgery.

We divided the length of history into three groups: 0–14 days; 15–31 days; more than 31 days. In the length of the history of more than 31-day group, only 4 out of 83 patients failed to insert 14F UAS. A longer history may reduce the failure rate by about 80% (length of history >31 days VS 0–14 days, odds ratio: 0.202 and 95% CI [0.051, 0.805], *p*=0.023, Figure 2). The reason for the differences between the groups was unknown. Some drugs such as alpha-blockers and anti-inflammation therapy before surgery might be beneficial in facilitating UAS placement [11, 15]. Due to the lack of detailed data, medical therapy history and the duration before the RIRS were not evaluated in this research. Research focused on the relationship between the diameter of the ureter, and vascularization has not ever been conducted. Here, we found that the diameter of the ipsilateral common iliac artery was another factor that was associated with the success of UAS placement. Computed tomography (CT)-based measurements contained seven parameters explored in this study, and most of the parameters were meaningless for predicting the UAS placement. We measured the diameter of the artery because it is a luminal structure like the ureter, and the artery is adjacent to the ureter, as well as it is unlike a vein which may change with blood volume and so on. We hypothesized that the diameter of the artery might be related to the diameter of the ureter. Fortunately, the long diameter of the ipsilateral common iliac artery (odds ratio: 1.285 and 95% CI [1.018, 1.623], *p*=0.035) was found to be facilitated with UAS placement (Figure 2). The correlation between the diameter of the artery and the ureter may be related to individual growth and development and may also be related to the crossing of the ureter across the iliac blood vessel, where the ureter crossing the iliac blood vessel is one of its three stenosis sites. It was noted that the odds ratio value was not high enough, and its predictions had limited value. What is more, this index was not found to be associated with the success of UAS placement in subgroup analysis. We expected that some other indexes would be included in subgroup analysis, such as ureter diameter above ureteral calculi, the degree of hydronephrosis, and others.

To sum up, we found females with a longer length of history and a longer diameter of the ipsilateral common iliac artery were beneficial for the success of UAS placement. These factors were easy to be determined before surgery and may be helpful for clinical decisions. We recommend using smaller UAS for these groups or we should insert a ureteral stent and leave it for two weeks in these patients with risk factors of failing to insert UAS. However, our study is not devoid of limitations; first, the study is retrospective in nature, which may lead to selection bias, and we tried to overcome this limitation by including all cases in the period; second, the data was from one institution in China, and the results were not widely representative; third, the patient's preoperative medication was not recorded, which may be associated with the success of UAS placement, and urine factors (e.g.; inflammatory factors which can influence ureteral epithelium) were not evaluated. We look forward to a large sample of multicenter studies to confirm our conclusions.

## 5. Conclusion

In conclusion, about 11.2% of patients failed to insert the ureteral access sheath (UAS), and our data indicated males, a short length of history, and a short diameter of the ipsilateral common iliac artery were the risk factors for the failure of UAS placement.

## Figures and Tables

**Table 1:** Characteristics of included patients.

Parameters	Failed to insert UAS (*n* = 29)	Success to insert UAS (*n* = 231)	*P* value
Age (years), mean (SD)	42.4 ± 13.4	47.3 ± 13.1	0.056
Gender (males), *n* (%)	23 (79.3%)	140 (60.6%)	0.050
Height (cm), mean (SD)	167.00 ± 8.35	164.59 ± 7.96	0.128
Weight (kg), mean (SD)	68.33 ± 13.51	65.29 ± 11.40	0.186
BMI (kg/m^2^)	24.31 ± 3.41	23.99 ± 3.14	0.606

Stone side			
Right	15	109	0.645
Left	14	122	

Stone location			
Kidney	6	70	0.283
Ureter	23	161	

Hydronephrosis			
Yes	22	160	0.465
No	7	71	

Length of history			
0–14 days	19	118	0.083
15–31 days	6	34	
>31 days	4	79	

CT based parameters			
Long diameter of ureteral calculi (mm), mean (SD)	10.79 ± 5.56	10.95 ± 4.83	0.873
Short diameter of ureteral calculi (mm), mean (SD)	7.62 ± 3.45	7.56 ± 2.22	0.926
Diameter of the widest part of the kidney parenchyma (mm), mean (SD)	23.24 ± 4.71	21.95 ± 5.09	0.195
Diameter of the narrowest part of the renal parenchyma (mm), mean (SD)	12.62 ± 3.28	11.95 ± 3.66	0.346
Diameter of the abdominal aorta (mm), mean (SD)	15.4 ± 2.2	15.7 ± 2.2	0.559
Diameter of the ipsilateral common iliac artery (mm), mean (SD)	10.5 ± 1.8	11.2 ± 2.2	0.071
Diameter of the contralateral common iliac artery (mm), mean (SD)	10.6 ± 3.2	11.5 ± 2.8	0.122

UAS: ureteral access sheath.

## Data Availability

The data used to support the findings of this study are available from the corresponding author upon request.
